# Extracellular and macropinocytosis internalized ATP work together to induce epithelial–mesenchymal transition and other early metastatic activities in lung cancer

**DOI:** 10.1186/s12935-019-0973-0

**Published:** 2019-10-01

**Authors:** Yanyang Cao, Xuan Wang, Yunsheng Li, Maria Evers, Haiyun Zhang, Xiaozhuo Chen

**Affiliations:** 1 Department of Biological Sciences, Ohio University, Athens, OH 45701 USA; 2 Interdisciplinary Graduate Program in Molecular and Cellular Biology, Ohio University, Athens, OH 45701 USA; 3 The Edison Biotechnology Institute, Ohio University, Athens, OH 45701 USA; 4 Honors Tutorial College, Ohio University, Athens, OH 45701 USA; 5 Department of Chemistry and Biochemistry, Ohio University, Athens, OH 45701 USA; 6 Department of Biomedical Sciences, Heritage College of Osteopathic Medicine, Ohio University, Athens, OH 45701 USA

**Keywords:** Tumor microenvironment, Invasion, EMT, ATP internalization, TGF-β

## Abstract

**Background:**

Extracellular ATP (eATP) was shown to induce epithelial–mesenchymal transition (EMT), a very important early process in metastasis, in cancer cells via purinergic receptor signaling. However, the exact induction mechanisms are far from fully known. We previously described that eATP is internalized by cancer cells in vitro and in vivo by macropinocytosis in human non-small cell lung cancer A549 and other cancer cells, drastically elevates intracellular ATP levels, enhances cell proliferation and resistance to anticancer drugs. In this study, we tested the hypothesis that eATP and macropinocytosis-internalized eATP also induces EMT and other early steps of metastasis.

**Methods:**

Floating cells, fencing, and transwell assays were used to show that ATP induces cell detachment, new colony formation, migration and invasion in human A549 and other lung cancer cells. Western blots were used to detect ATP-induced changes in EMT-related proteins; Confocal microscopy was used to demonstrate ATP-induced metastasis-related cell morphological changes. Inhibitors and siRNA knockdowns were used to determine P2X7’s involvement in the ATP-induced EMT. CRISPR–Cas9 knockout of the SNX5 gene was used to identify macropinocytosis’ roles in EMT and cancer cell growth both in vitro and in vivo. Student t-test and one-way ANOVA were used to determine statistical significance, P < 0.05 was considered significant.

**Results:**

eATP potently induces expression of matrix metallopeptidases (MMPs), and detachment, EMT, migration, and invasion of lung cancer cells. The induction was independent of TGF-β and semi-independent of P2X7 activation. eATP performs these functions not only extracellularly, but also intracellularly after being macropinocytically internalized to further enhance P2X7-mediated EMT, filopodia formation and other early steps of metastasis. The knockout of macropinocytosis-associated SNX5 gene significantly reduces macropinocytosis, slows down tumor growth, and changes tumor morphology in nude mice.

**Conclusions:**

Collectively, these results show that eATP's functions in these processes not only from outside of cancer cells but also inside after being macropinocytotically internalized. These findings shed light on eATP’s initiator and effector roles in almost every step in early metastasis, which calls for rethinking and rebalancing energy equations of intracellular biochemical reactions and the Warburg effect, and identifies eATP and macropinocytosis as novel targets for potentially slowing down EMT and preventing metastasis.

## Background

Metastasis is estimated to be responsible for more than 90% of all cancer related death [1, 2]. However, due to its complexity, it remains the least understood in cancer biology. The early steps of metastasis involve increased proteolysis, loss of cell–cell adhesion, migration, and invasion of primary tumor cells into surrounding normal tissues. These steps are initiated and accompanied by the induction of epithelial–mesenchymal transition (EMT) in a wide variety of cancer. In those cancers, EMT is essential for the high motile and invasive characteristics of cancer cells [3, 4], and involves turning on genes for generation of mesenchymal cell phenotypes and turning off genes for epithelial cell characteristics, although EMT is rarely complete. Ample experimental evidence indicates that tumor cells undergo partial (incomplete) EMT, when they express a mixture of E or M markers at different levels at different times of the induction. Being partially induced, these tumor cells show the maximal tumor-initiating capacity and are considered to be staying in a metastable phenotypic state [5, 6]. These changes set the stage for cancer cells to move from their original locations to new places within the primary tumors (migration) or invade into surrounding normal tissues before moving on to distant organs (metastasis) [7, 8]. During induction, a key early EMT-marker and major cellular adhesion molecule in the tight adhesion junction, E-cadherin, is cleaved by MMP proteases. MMPs directly modulate the detachment and migration by cleaving cell–cell or cell–matrix adhesion molecules and/or by degradation of extracellular matrix (ECM), both at the primary tumor site and the secondary colonization site [9]. Losing the junction, individual tumor cells change their morphology, grow filopodia-like protrusions, and then migrate within the tumor or invade into surrounding normal tissues and blood vessels, initiating metastases [3, 4, 7, 8]. EMT is a group of cell-biological programs that is also regulated at the gene expression level by a series of master EMT-inducing transcription factors (EMT-TFs), including Snail, Slug, and others [10, 11]. However, how EMT induction is triggered and regulated is not fully known.

In both normal tissues and in cancer, one major EMT inducer is TGF-β [12, 13], which binds to cell membrane associated TGF-β receptor, triggering a cascade of signaling events which leads to exocytosis of ATP-containing vesicles [12, 13]. Once released, extracellular ATP (eATP) binds to a purinergic receptor, P2X7, activating the P2X7-mediated signaling pathway, eventually resulting in EMT induction [12–15]. However, the exact functional relationship between ATP and TGF-β in EMT induction is not fully known. Furthermore, the roles of eATP in the P2X7 activation and signaling are not presently fully understood.

Intratumoral extracellular ATP concentrations have been found to be 10^3^–10^4^ times higher than those found in normal tissues [16–19], in the range of 200 to more than 500 μM. It is not completely clear where the eATP goes to and how it is used. We were the first to report that eATP is internalized in various cancer cells primarily by macropinocytosis and other endocytoses, both in vitro and in vivo [20, 21]. The internalized eATP greatly elevates intracellular ATP (iATP) concentration, promotes cell growth rate, and enhances cell survival [20, 21]. More recently, we reported that eATP also substantially enhances resistance to chemo and target drugs in 5 different cancer types studied [22]. Our findings on eATP’s new functions, particularly those performed with ATP internalization mediated by macropinocytosis, have recently been reviewed [23], impacting the current view on roles of eATP and macropinocytosis in tumorigenesis, cancer drug resistance, and the Warburg effect.

Based on all these observations made by us and others, we hypothesized that eATP, working from both outside and inside of cancer cells, induces EMT and other early steps of metastasis, such as cell migration and invasion. To test this hypothesis, we performed various in vitro and in vivo assays to determine if eATP, working alone, induces cancer cells’ (i) detachment, (ii) EMT, (iii) increase in cell migration and invasion, (iv) afore-mentioned activities from both outside and inside of cancer cells independently of TGF-β and dependently of macropinocytosis. CRISPR–Cas9 technology was used to knock out a key macropinocytosis-related gene, *SNX5,* to evaluate its role in eATP induced activities both in vitro and in vivo. The results of these studies show important previously-unrecognized contributions made by eATP in EMT and metastasis induction and profound implications in reconsidering energy (ATP) synthesis, supply and usage in cancer cells, and blocking cancer metastasis progression by targeting eATP and macropinocytosis.

## Materials and methods

### Chemicals and antibodies

DMEM was purchased from Corning. FBS was purchased from ATCC. ATP (adenosine 5′-triphosphate), suramin, BAPTA, oATP and KN62 were purchased from Sigma-Aldrich. Alexa Fluor™ 488 Phalloidin was purchased form Thermo Fisher Scientific. Antibody against E-cadherin, β-Catenin, ZO-1, N-cadherin, Vimentin, Snail, Slug, Twist, P2X7 and β-actin were purchased from Cell Signaling. Rabbit anti-SNX5 antibody was purchased from Abcam.

### Cell lines and cell culture

Human non-small cell lung cancer (NSCLC) cell lines A549, HOP-92, and H1299 were purchased from ATCC. A549 cells were cultured in Dulbecco’s Modified Eagle Medium (DMEM contains 25 mM glucose) supplemented with 10% fetal bovine serum, 50 I.U./ml penicillin, and 50 μg/ml streptomycin. H1299 and HOP-92 cells were cultured in RPMI 1640, supplemented with 10% fetal bovine serum, 2 mM l-glutamine, 50 I.U./ml penicillin, and 50 μg/ml streptomycin. All cells were grown in a humidified atmosphere of 5% CO_2_ at 37 °C.

### Floating cell counting and clonogenic assay

Cells were cultured in 24-well plates overnight following treatment with 0, 0.5 and 1.0 mM ATP in triplicate at 37 °C. Floating cells were collected from each condition at a different time point. Then floating cells were recovered by centrifugation at 200–300 g (1100 rpm on table top centrifuge) for 5 min at room temperature, the cell pellets were re-suspended in cell growth medium. The cell suspension was diluted 1:1 with 0.4% trypan blue and viable floating cells were counted with a hemocytometer under bright-field microscopy (200× magnification).

For clonogenic assays, 4 h after the treatment with or without ATP, floating cells were collected from the same volume medium and seeded in 100 mm cell culture dish. All conditions were in triplicate. Cells were cultured in DMEM supplemented with 10% fetal bovine serum for 2 weeks. The cells were then washed three times with PBS and fixed with 4% formaldehyde for 15 min at room temperature. After fixation, cells were stained with 1% crystal violet in methanol for 15 min, then rinsed with distilled water and dried. Colonies with sizes over 0.5 mm were counted. The numbers of counted colonies from each triplicate plate were averaged and compared to the average number of colonies in the non-ATP treated group.

### Fence assay

To study how floating cells form new colonies at new locations, cells were seeded inside a ring-shaped plastic device (2.5 × 10^4^ cells/200 μl/well), which was placed in the center of a 60 mm cell culture dish. After cell attachment, the ring was removed and the cells were treated with or without ATP for 14 days. Floating cells were now able to settle at any part of the dish away from the central ring. Each treatment condition was in triplicate. The dishes were then washed with PBS; the cells were fixed with 4% formaldehyde for 15 min at room temperature and stained with 1% crystal violet in methanol for 15 min. The numbers of individual colonies were photographed and counted by scanner.

### Cell migration assay

Cell migration rates were measured by the in vitro wound healing assay. Cells were seeded into 6-well plates and grown to confluence. Confluent cell monolayers were scratched (wounded) by a sterile micropipette tip to generate a gap without cells, and the wounded monolayers were washed three times with PBS to remove cell debris. Wounded cells were further incubated with or without 0.5 or 1 mM ATP for 12–24 h. The remaining gaps were photographed at 0, 12 and 24 h after wounding. The migration rates were evaluated by measuring the width of the wounds at different time points and compared with those of the non-ATP treated samples.

Cell migration capacity was also determined by using 24-well cell culture Transwell chambers (6.5 mm Transwell with 8.0 µm pore polycarbonate membrane, Corning). The upper insert was seeded with A549 or HOP-92 cells (2 × 10^4^ cells/200 µl/well) in serum-free DMEM, and the lower inserts were containing DMEM with 10% FBS. The cells were incubated with or without ATP. After 16 h incubation at 37 °C, cells were fixed with paraformaldehyde for 15 min at room temperature and stained with crystal violet for 15 min at room temperature. Non-migrated cells on the upper surface were removed using cotton swabs. The numbers of migrated cells were counted from six randomly selected visual fields using compound light microscopy (200× magnification).

### In vitro invasion assay

The invasive ability of NSCLC cells was evaluated using Transwell Chamber invasion assay (Corning). The procedure was identical to Transwell migration assay except that the polycarbonate filter was coated with reconstituted basement membrane Matrigel, and ATP treatment time. The cells were incubated with or without extracellular ATP at various concentrations (0.1–1.0 mM) for 20 h at 37 °C. After the cell wash, fixation, and staining, the number of invasive cells, which “eat through the Matrigel” to reach the other side were counted by compound-light microscopy at 200× magnification. The average number of invasive cells was determined from six randomly selected visual fields.

### Purinergic receptor signaling study

A549 cells in DMEM supplemented with 1 mM ATP were seeded in Transwell chambers and treated with suramin, a PR inhibitor targeting P2 receptors, and BAPTA, a Ca^2+^ chelator that blocks general PR signaling or P2X7 inhibitor (KN-62 or oxidized ATP). After 20 h of incubation, the invasive cells were counted as described above in “In vitro invasion assay” section.

### Confocal immunofluorescence microscopy

To observe changes of cell protrusion such as filopodia in cells, F-actin (filamentous-actin) of cells were stained with Fluorescent Phallotoxins. The A549 or H1299 cells were seeded overnight on glass coverslips placed in 6-well plates, then treated with or without ATP. Before staining, cells on coverslips were fixed with 4% formaldehyde solution in PBS at room temperature for 10 min and permeabilized with 0.1% Triton X-100 in PBS for 5 min. After washings with PBS, fixed cells were incubated with 150 nM Alexa Fluor™ 488 Phalloidin (Thermo Fisher Scientific) dissolved in PBS for 20 min. Counterstaining with ProLong Gold Antifade Mountant (Thermo Fisher Scientific) was used to visualize and verify the location of the nucleus. Stained cells were examined and photographed using a Confocal Fluorescence Microscope (A1R, Nikon) at 1000× magnification.

### RNA extraction and RT-qPCR

Total RNA from A549 cells was extracted by an RNA purification kit (Thermo Fisher) following manufacturer’s instruction. Total RNA (1 μg) was reverse transcribed using a cDNA synthesis kit (Thermo Fisher). cDNA was amplified using SYBR Green qPCR Master Mix (Thermo Fisher) on StepOne Real-Time PCR System (Applied Biosystems). The primers targeted MMP7, MMP9, MMP13 and β-actin are listed in Table 1. Thermal cycle condition was: 95 °C, 10 min; (95 °C, 15 s) × 40; 95 °C, 15 s; 60 °C, 30 s. The expression of targeted gene were normalized by β-actin and quantified using the 2^−ΔΔCt^ method.

**Table 1:** Real-time (RT) PCR primers

Gene	Primer sequence
MMP7	Forward: 5′-AAAATGGACTTCCAAAGTGG-3′
	Reverse: 5′-AAAGCCTTTGACACTAATCG-3′
MMP9	Forward: 5′-AGCTGGCAGAGGAATAC-3′
	Reverse: 5′-CCCCAGAGATTTCGACTC-3′
MMP13	Forward: 5′-AGGCTACAACTTGTTTCTTG-3′
	Reverse: 5′-AGGTGTAGATAGGAAACATGAG-3′
β-actin	Forward: 5′-GACGACATGGAGAAAATCTG-3′
	Reverse: 5′-ATGATCTGGGTCATCTTCTC-3′

### Protein analysis

Proteins were isolated from cells treated with or without ATP. Proteins were analyzed with western blots using appropriate primary antibodies: E-cadherin (Rabbit, 1:1000, CST, #3195), β-Catenin (Rabbit, 1:1000, CST, #8480), ZO-1 (Rabbit, 1:1000, CST, #8193), N-cadherin (Rabbit, 1:1000, CST, #13116), Snail (Rabbit, 1:1000, CST, #3879), Slug (Rabbit, 1:1000, CST, #9585), Vimentin (Rabbit, 1:1000, CST, #5741), SNX5 (Rabbit, 1:1000, Abcam, ab180520), P2X7 (Rabbit, 1:500, CST, #13809). Secondary antibody staining was completed with anti-rabbit IgG, HRP-linked antibody (Goat, 1:1000, CST, #7074). β-actin was used as a protein loading control. The signals were detected with Super Signal West Pico Chemiluminescent substrate (Thermo Fisher Scientific) and exposed to film. Quantification of Western blots was performed by densitometry analysis with software ImageJ (NIH).

### Small interfering RNA (siRNA) study

Small interference RNA (siRNA) for P2X7, SNX5 and negative control (scrambled) siRNA were purchased from Santa Cruz, Qiagen and Thermo Fisher Scientific, respectively. P2X7 siRNA is a pool of three target-specific 19–25 nt siRNAs. The targeting sequences for SNX5 siRNA is 5′-ACAGGTATATATGGAAACAAA-3′. A scramble sequence not targeting any known gene was used as a negative control. siRNA transfection was performed using Lipofectamine RNAiMAX transfection reagent (Thermo Fisher Scientific) according to the manufacturer instructions. Briefly, A549 cells were seeded at 30–50% confluence in 96-well or 6-well plates in DMEM. The following day, a pre-incubated mixture of 20 nM siRNA (P2X7, SNX5 or scrambled siRNA) and Opti-MEM I reduced serum medium (Invitrogen) at 1:1 ratio was added to the cultured cells. The cells were incubated at 37 °C for 48 h. The knockdown efficiency was determined by western blot using anti-SNX5 antibody (Abcam) or anti-P2X7 receptor antibody, and the cells were used for ATP assay, Transwell assay and EMT-associated protein level analysis.

### ATP assay

Intracellular ATP levels were measured using luminescence ATP detection assay system (PerkinElmer) according to manufacturer’s instructions and as described previously [20–22].

### Immunofluorescence

A549 cells were seeded on glass coverslips treated with 0.5 or 1 mM ATP for 24 h and then fixed in 4% paraformaldehyde for 15 min at room temperature. The cells were then blocked with 0.3% Triton X-100 and 5% goat serum in PBS for 1 h. After washing with PBS, the cells were incubated with primary antibodies at 4 °C overnight. Primary antibodies are E-cadherin (1:200, Cell Signaling), Vimentin (1:200, Cell Signaling). After incubation, the cells were washed and incubated with Alexa 488-conjugated or Alex-594-conjugated secondary antibody for 1 h. Coverslips were counterstained with ProLong Gold Antifade Mountant with DAPI (Thermo Fisher Scientific). The fluorescence images were taken using a fluorescence microscope (ECLIPSE E600, Nikon).

### CRISPR–Cas9 mediated gene knockout

CRISPR guide RNA (gRNA) sequence design and plasmid preparation were provided by GenScript (Piscataway, NJ). The 20-nucleotide target sequences of SNX5 CRISPR guide RNA is 3′-CAAATTTACAGTGCACACAA-5′. *SNX5* targeting CRISPR–Cas 9 plasmid was transfected into A549 cells using Lipofectamine 3000 Transfection Reagent (Thermo Fisher Scientific) according to the manufacturer’s instruction. 48 h after transfection, cells were refreshed with growth media plus 1.5 µg/ml puromycin for drug selection for 3 days. Resistant cells were plated at single cell dilutions into 96-well plates for clonal expansion. After 10–14 days, colonies were tested for *SNX5* knockout by western blot and other functional assays. The homogeneous cell populations (double allele KO cells) were selected for further study.

### In vivo tumor studies

Male nude mice of Nu/Nu strain of 5 weeks of age were purchased from the Jackson Laboratory (Bar Harbor, ME) and maintained under specific pathogen-free conditions.

A549 or SNX5 knockout (KO) A549 (A549snx5ko) cells were injected subcutaneously into the flank of male Nu/Nu mice of 6 weeks of age at 5 × 10^6^ cells per injection, nine mice per group (N = 9). Tumor size was measured using digital calipers twice a week, and tumor volume was calculated as (length × width × width)/2 in mm^3^. 5 weeks after cell injection, mice were euthanized and tumors were surgically removed, weighed, and photographed for comparison.

All animal studies were done in accordance to US government regulation on animal care and Ohio University IACUC approved protocol.

### Statistical analysis

Each experimental condition was performed in triplicate or hexads and repeated at least once. Results were reported as mean ± standard deviation. The statistical difference, or difference between control and other groups was analyzed using Student’s t-test or one-way ANOVA with GraphPad Prism 7.0 software. P < 0.05 was considered statistically significant. *, P < 0.05, **, P < 0.01 and ***, P < 0.001.

## Results

### ATP induced cell detachment and formation of new colonies distant from their original places

**Fig. 1:**
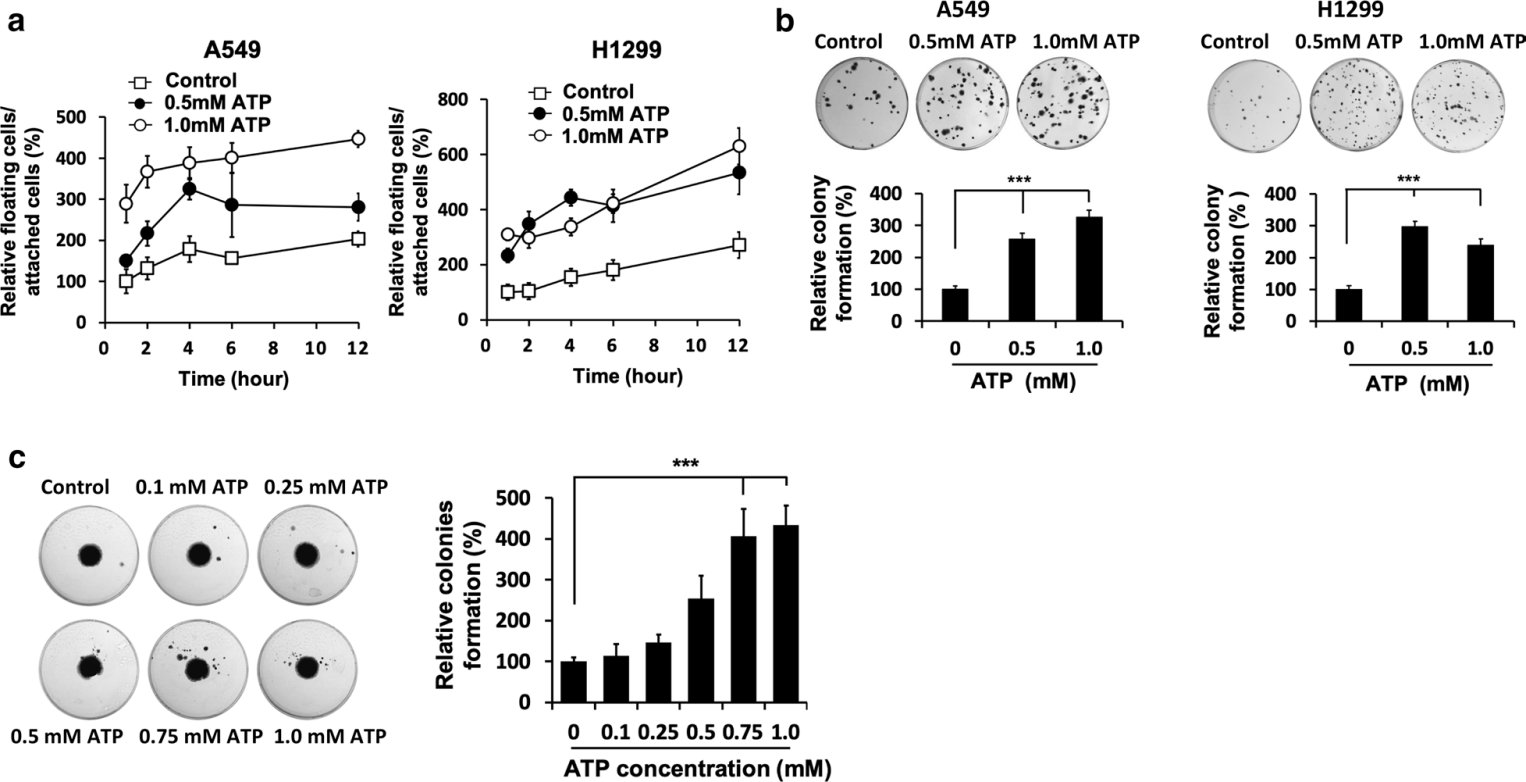
Extracellular ATP induces detachment and recolonization of cancer cells. A549 or H1299 cells growing in cell culture plates were incubated with or without ATP for various times. After incubation, floating cells were collected, and the live floating cells were counted. The total floating cells were then re-plated for clone formation. **a** Direct count of viable floating cells under different ATP concentration. **b** A clonogenic assay using floating cells isolated from the same volume of cell culture media. **c** Fence assay. Cells were seeded inside a ring-shaped plastic device. After cell attachment, the ring was removed, and the cells were incubated with or without ATP for 2 weeks for examination of cell detachment and recolonization at other places in the wells. Experiments were performed in triplicate and data are presented as the mean ± standard deviation. *P < 0.05, **P < 0.01 and ***P < 0.001

We first determined if eATP induces cell detachment. Treatment of A549 or H1299 cells with either 0.5 mM or 1 mM ATP resulted in an increase of floating cells in the cell culture dish within 1 h (Fig. 1a). These ATP concentrations were those found in tumors [16–19]. 1 mM ATP in general resulted in more floating cells than 0.5 mM ATP, and this phenomenon appeared to reach a plateau at around 4 h. These changes provide mechanistic explanations for the observation of cell detachment and cell floating after ATP treatment (Fig. 1a, b). We confirmed the viability of the floating cells by a clonogenic assay (Fig. 1b). The fence assay revealed that the detached cells formed new colonies at areas distant from the original center region and the number of new colonies was ATP concentration dependent (Fig. 1c), most likely via the route of floating cells. These results show eATP induces cell detachment leading to recolonization of floating cells distant from the original plating site.

### ATP induced cell migration and invasion

**Fig. 2:**
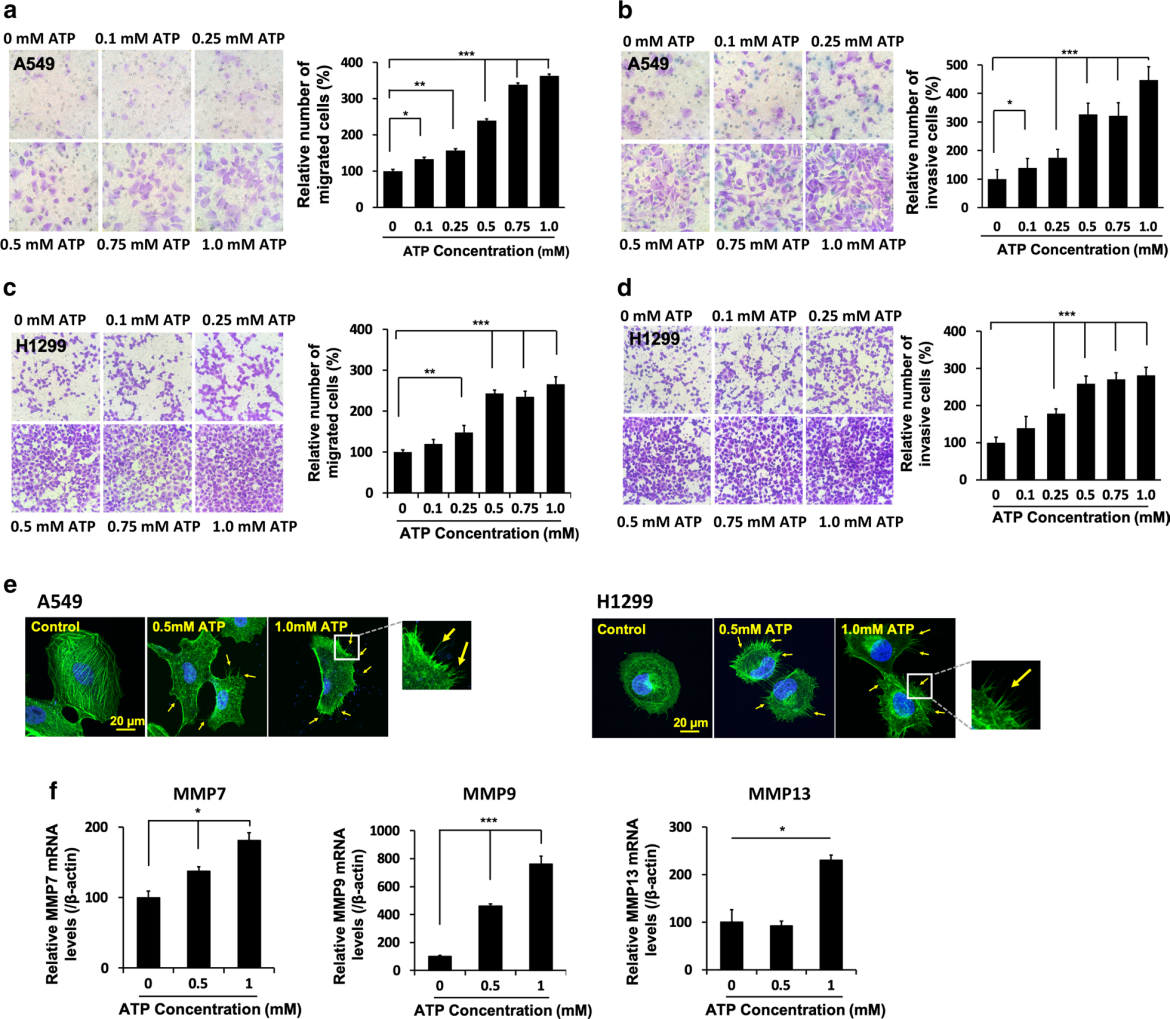
Extracellular ATP enhances the motility, invasion, MMPs expression and alters cell morphology in NSCLC cells. **a**–**d** Effect of ATP on the migration and invasion of A549 and H1299 cells using Transwell assays. Cells treated with various concentrations of ATP in migration assay (16 h) or invasion assay (24 h). Cells were then stained with crystal violet and observed under the microscope with × 200 magnification. Representative images and the quantitative analysis following **a**, **b** the migration and invasion assays in A549 cells. **c**, **d** the migration and invasion assay in H1299 cells. **e** Effect of extracellular ATP on EMT-related morphological changes of A549 and H1299 cells. A549 and H1299 cells after 24 h incubation with ATP (0.5 mM or 1.0 mM) or with vehicle (control) were fixed and stained with Alexa Fluor^®^ 488 Phalloidin (green) and DAPI (blue). The images were examined and photographed using confocal microscopy. Arrowheads show the formation of F-actin-enriched membrane protrusions. **f** Dose dependent effect of ATP on mRNA levels of MMP7, MMP9 and MMP13 in A549 cells. Experiments were performed in triplicate and data are presented as the mean ± standard deviation. *P < 0.05, **P < 0.01 and ***P < 0.001

After demonstrating eATP’s capability of inducing cell detachment, we went on to determine if eATP promotes cell migration and invasion. In a wound healing assay, ATP treatment resulted in faster “healing” or cell movement at both 12 and 24 h (Additional file 1: Figure S1a, b). In Transwell assays, ATP induced a dose-dependent increase in cell migration (Fig. 2a) and cell invasion (Fig. 2b) for not only A549 cells but also for H1299 (Fig. 2c, d) and Hop92 cells (Additional file 1: Figure S1c, d), indicating that the ATP-induced migration and invasion activities are present in all three human lung cancer cell lines tested.

### Extracellular ATP induced formation of filopodia-like protrusions

Confocal microscopy revealed that eATP treatment induced formation of F-actin-enriched filopodia-like protrusions on plasma membrane in A549 and H1299 human lung cancer cells (Fig. 2e), an indication of EMT and a key cell morphological change required for cell migration and invasion. Because of the similar phenotypic changes induced by eATP in different cell lines, subsequent assays were primarily done in A549 cells as a representative lung cancer cell type.

### Extracellular ATP induced the expression of matrix metallopeptidase (MMPs)

To evaluate whether extracellular ATP alters the expression of matrix metallopeptidase (MMPs), RT-qPCR was used to analyze mRNA levels of MMP7, 9 and 13 in A549 cells. Compare to the control, the expression of MMP7 was increased to 1.4- and 1.8-fold by the treatment of 0.5 mM and 1.0 mM ATP, respectively, and that of MMP9 was increased to 4.6-, and 7.6-fold, respectively. The expression of MMP13 was increased to 2.3-fold in 1.0 mM ATP treated compared to the control group (Fig. 2f). These changes provide mechanistic explanations for the observation of cell detachment, migration, and invasion after ATP treatment (Figs. 1a, 2a–d).

### Purinergic receptor (PR) inhibitors and PR siRNA knockdown reduced cell migration

**Fig. 3:**
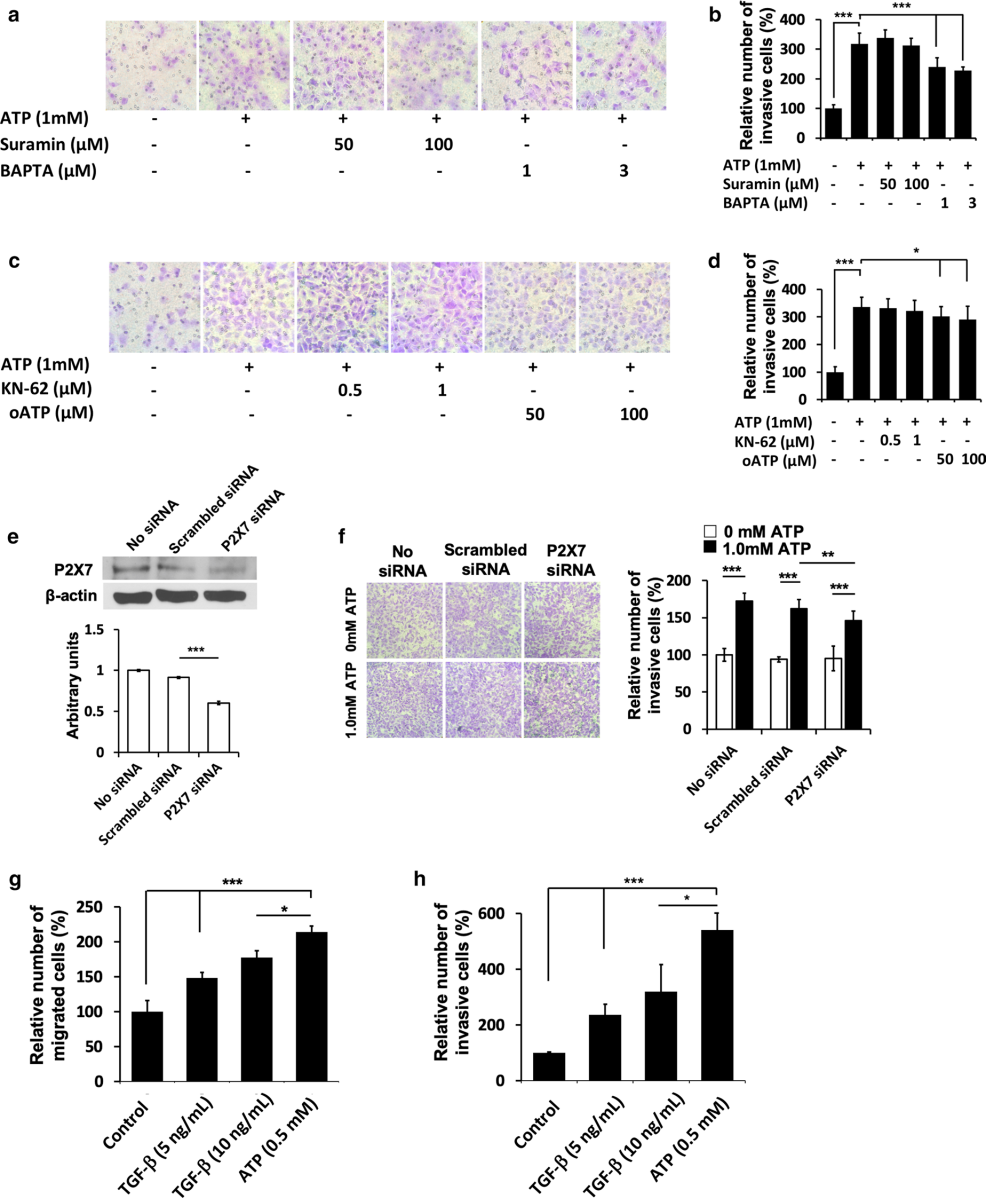
Purinergic Receptor and TGF-β signaling in ATP-induced invasion in A549 cells. Effect of non-selective P2 purinergic antagonist (suramin and BAPTA) and P2X7 inhibitors (KN-62 and oATP), on A549 cell invasion. A549 cells were incubated with different inhibitors in the presence and absence of extracellular ATP for 20 h, and differential invasion rates were determined using Transwell assays. **a**, **b** Representative images and quantification of the invaded cells under suramin and BAPTA treatment. **c**, **d** Representative images and quantification of invaded cells under KN-62 and oATP treatment. **e** A549 cells were transfected with scrambled siRNA or *P2X7* siRNA and incubated for 48 h. The expression of *P2X7* was detected by Western blot. **f** 48 h after transfection, ATP-induced cell invasion was examined using a Transwell Assay. **g** Relative effects of ATP and TGF-β on cell migration. **h** Relative effects of ATP and TGF-β on cell invasion. Experiments were performed in triplicate and data are presented as the mean ± standard deviation. *P < 0.05, **P < 0.01 and ***P < 0.001

Extracellular ATP was known to induce PR signaling [11–13] and eATP induced P2X7 signaling has been shown to be involved in TGF-β-mediated EMT induction [12, 13]. When general PR inhibitors were used to treat A549 cells, Suramin was found to have no effect on cell migration while BAPTA significantly reduced cell migration (Fig. 3a, b). When specific PR inhibitors were used, KN62 showed insignificant reduction, while oxidized ATP (oATP) showed significant reduction in cell migration (Fig. 3c, d). Furthermore, knockdown with P2X7 specific siRNA significantly reduced P2X7 protein levels (Fig. 3e) and cell invasion (Fig. 3f). These results indicate PR signaling, particularly P2X7 signaling, is involved in and at least partially responsible for eATP induced cell migration and invasion.

### Extracellular ATP can replace TGF-β for cancer cell migration and invasion

When ATP treated cells were compared with TGF-β treated cells, it was found that 0.5 mM ATP, a concentration in the range reported for intratumoral extracellular ATP levels [16–19], induced significantly more cell migration (Fig. 3g) and invasion (Fig. 3h) than TGF-β at two commonly used concentrations. This result indicates that eATP can replace TGF-β to independently induce the migratory and invasive activities of cancer cells, which are downstream of EMT.

### Extracellular ATP induced changes in expression levels of proteins involved in EMT

**Fig. 4:**
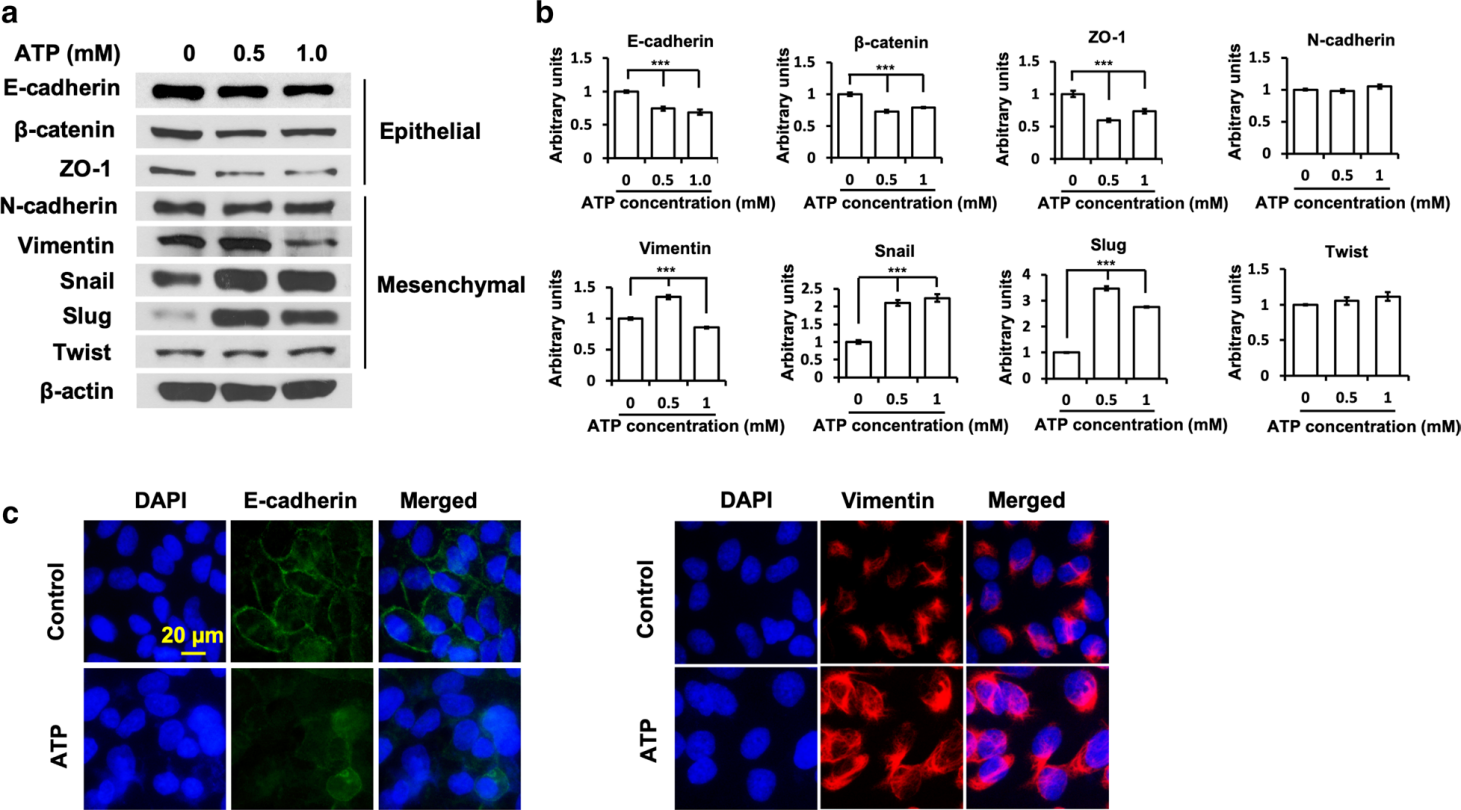
Extracellular ATP induces changes in levels and cellular distribution of proteins involved EMT. **a** A549 cells were treated with 0, 0.5 or 1.0 mM ATP for 24 h before analysis. EMT- associated proteins expression level was detected using western blot analysis. β-actin was used as a protein loading control for protein normalization. **b** Densitometry analyses of individual blots were performed using ImageJ software. **c** Immunofluorescence analysis of EMT-related proteins. Control and 0.5 mM ATP-treated A549 cells were fixed and stained with anti-E-Cadherin and vimentin antibodies observed by fluorescence microscopy with 400× magnification. Experiments were performed in triplicate and data are presented as the mean ± standard deviation. *P < 0.05, **P < 0.01 and ***P < 0.001

**Fig. 5:**
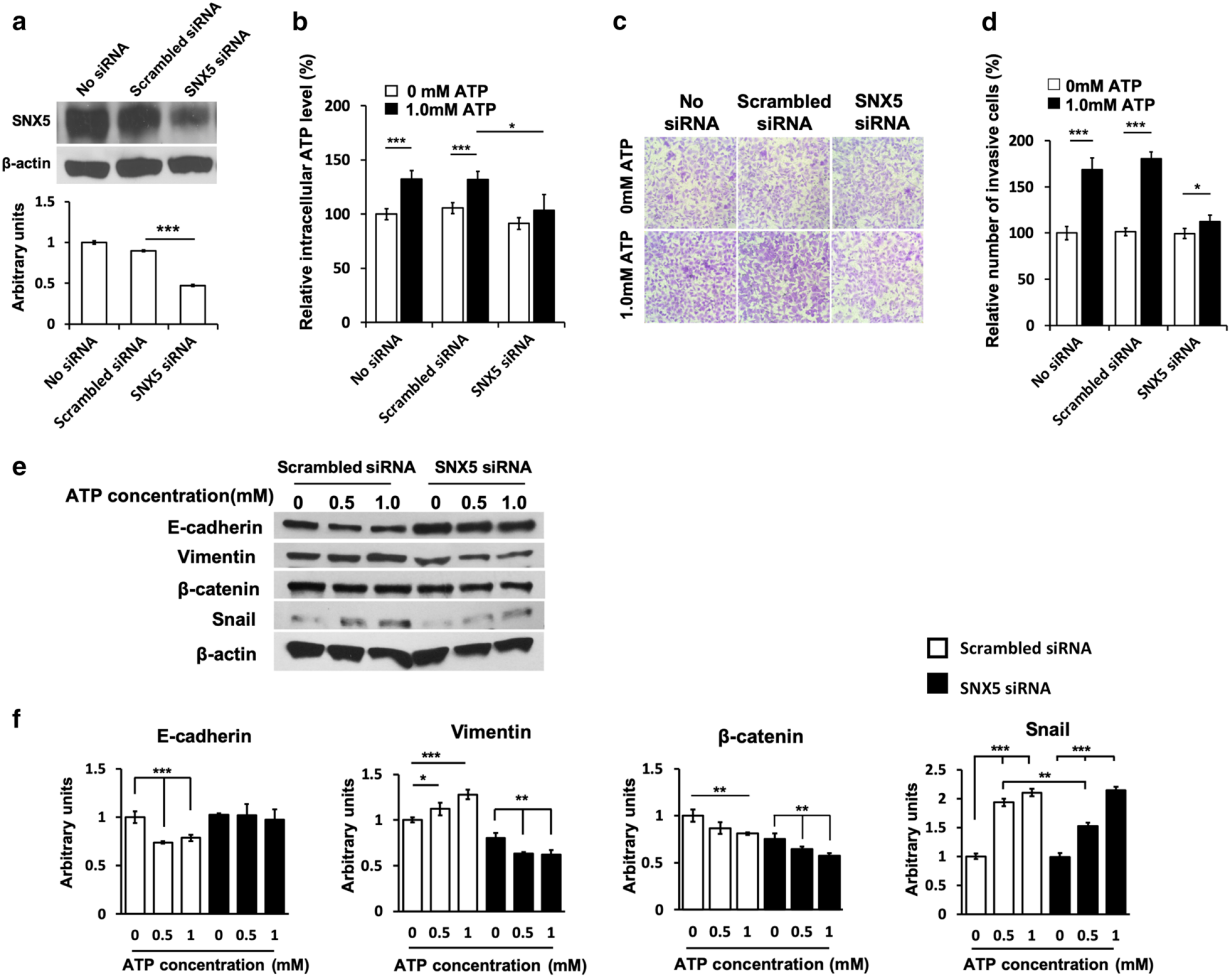
Involvement of macropinocytosis in extracellular ATP-induced migration and EMT induction. **a** A549 cells were transfected with scrambled siRNA or sorting nexin 5 (*SNX5*) siRNA and incubated for 48 h. The expression of SNX5 was detected by Western blot. **b** 48 h after transfection, cells were incubated with 0, 0.5 or 1.0 mM ATP and intracellular ATP levels were measured by ATP assay. **c**, **d** 48 h after transfection, ATP-induced cell invasion was examined using Transwell assays (**c**), and the quantitative analysis of invasive cells (**d**). **e** Control and *SNX5* knockdown cells were treated with 0, 0.5 or 1.0 mM ATP for 24 h before analysis. EMT-associated proteins: E-cadherin, vimentin, β-catenin and Snail protein expression levels were measured by western blot. **f** Densitometry analyses of individual blots were performed using ImageJ software. Experiments were performed in triplicate and data are presented as the mean ± standard deviation. *P < 0.05, **P < 0.01 and ***P < 0.001

Western blot analyses demonstrate that eATP induced protein level changes. The levels of most of the epithelial characteristic related proteins were reduced while some of the mesenchymal cell related proteins were elevated (Fig. 4a, b). Specifically, the expression of epithelial phenotype markers, E-cadherin, β-catenin and ZO-1 were reduced after ATP treatment. The mesenchymal-phenotype molecular and EMT-TFs, such as vimentin, Snail and Slug were upregulated at 0.5 mM ATP treatment. However, compared with 0.5 mM ATP treatment and no ATP controls, vimentin level showed decreases at 1 mM ATP treatment (Fig. 5a). The decrease may reflect a dose dependent effect on “partial EMT” [5] induced by eATP. Moreover, twist expression was not significantly changed in ATP treated group, suggesting transcription factor twist might not be involved in ATP-induced EMT process. These results were further confirmed by a fluorescence microscopy study targeting two representative EMT proteins E-cadherin and vimentin at whole cell level (Fig. 4c). The membrane localization E-cadherin was markedly decreased after incubated with ATP; whereas the vimentin was significantly increased in ATP-treated group. These results indicate that eATP induces changes in protein levels and cellular distributions of the proteins to activate EMT.

### siRNA knockdown of macropinocytosis related *SNX5* gene resulted in reduction of cell migration

Since eATP is internalized by A549 cells by macropinocytosis [20–22] and eATP might also promote EMT and cell migration/invasion intracellularly, we speculated that inhibition of macropinocysis might reduce them. Sorting nexin 5 (SNX5) is a protein very important for macropinocytosis and unrelated to other cellular functions such as cell movement [24, 25]. We decided to target the *SNX5* gene for demonstrating the involvement of macropinocytosis in eATP-induced intracellular changes and cell invasion. siRNA knockdown of the *SNX5* gene (Fig. 5a) resulted in reduction of intracellular ATP levels (Fig. 5b), indirectly indicating the reduction of macropinocytosis-mediated eATP internalization [20, 21]. The specific knockdown also led to reduction in cell invasion (Fig. 5c, d), and protein level restoration for epithelial cell related E-cadherin and reduction of mesenchymal cell associated vimentin and β-catenin with Snail being significantly changed at 0.5 mM of eATP (Fig. 5e, f). These results suggest that *SNX5* knockdown inhibited macropinocytosis, EMT induction, and cell migration and invasion, supportive to the hypothesis that macropinocytosis and internalized eATP contribute to EMT induction and cell migration and invasion.

### Knockout of *SNX5* gene resulted in reduced macropinocytosis, intracellular ATP levels, cell growth rate, and migration/invasion rates

**Fig. 6:**
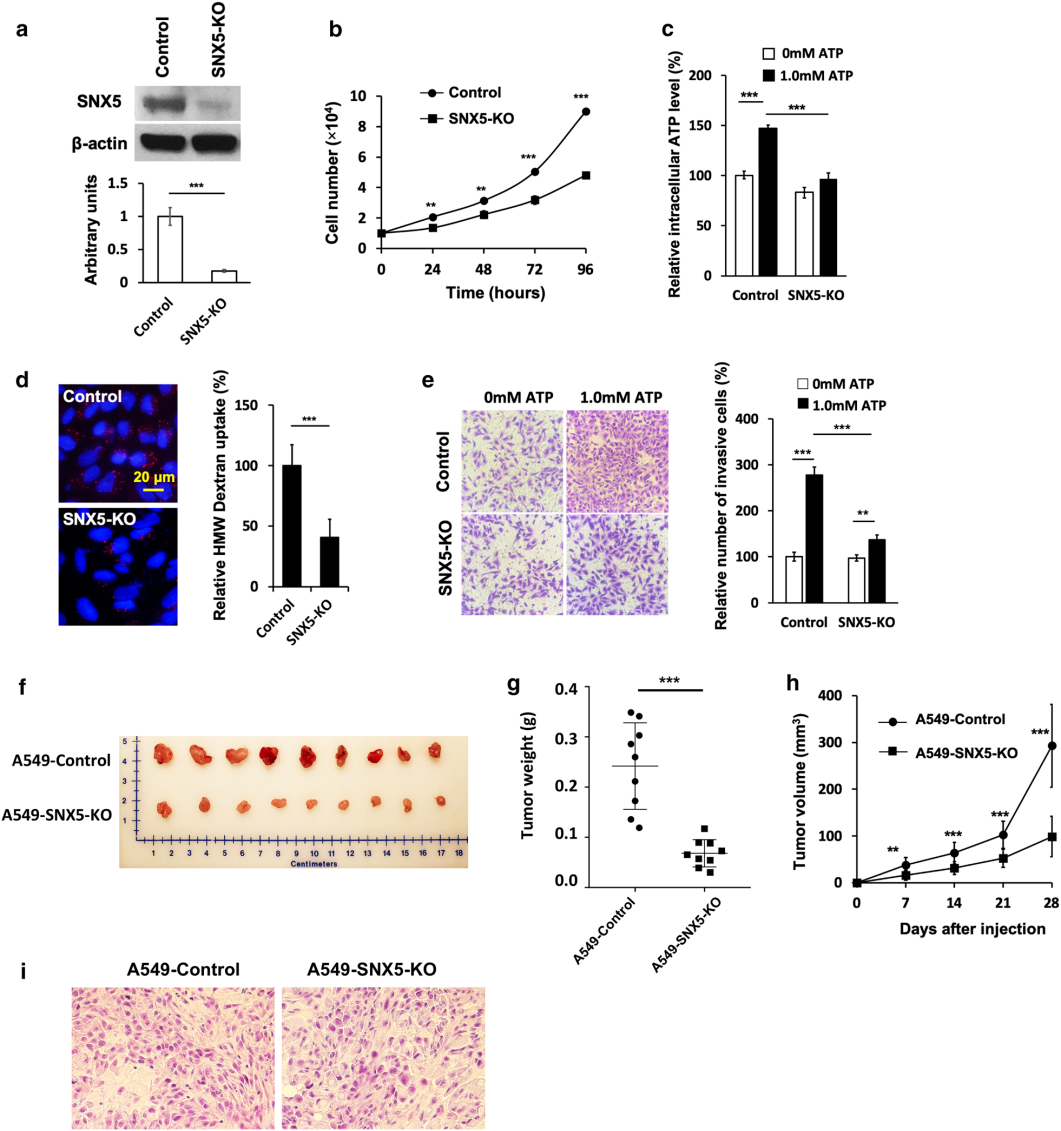
In vitro and in vivo effects of knocking out the *SNX5* gene in A549 cells. **a**–**e** CRISPR–Cas9 mediated *SNX5* knockout inhibits macropinocytosis, ATP internalization and suppresses migration/invasion. **a** Western blot confirmation for SNX5 protein knockout in A549 cells. **b** A direct count of viable cells of A549 control and A549snx5ko for determination of their respective cell proliferation rates. **c** Intracellular ATP levels of A549 (control) or A549snx5ko cells incubated with or without ATP, and the intracellular ATP levels were measured by ATP assay. **d** Fluorescence microscopy of A549 (control) and A549snx5ko cells treated with a known macropinocytosis tracer, TMR-dextran and quantification of intracellular TMR-dextran. **e** Transwell invasion assay results indicating the ATP-induced cell invasion ability of A549 and A549snx5ko cells, and the quantification of invasive cells. **f**–**h** A549 (control) or A549snx5ko cells were subcutaneously injected into flanks of male nude mice (n = 9 per group) to generate tumors. 4 weeks after inoculation, tumors were removed for examination. **f** Comparison of sizes of surgically removed tumors. **g** Tumor weights measured after surgical tumor removal. **h** Weekly tumor growth curves after tumor cell inoculation. **i** HE staining of A549 and A549snx5ko tumor sections. Experiments were performed in triplicate and data are presented as the mean ± standard deviation. *P < 0.05, **P < 0.01 and ***P < 0.001

To further assess the involvement and contribution of macropinocytosis in the eATP-induced EMT induction and metastasis, *SNX5* was knocked out (KO) by CRISPR–Cas9 technology. Multiple KO clones were selected by antibiotic treatment and serial cell dilutions and characterized for the loss of the *SNX5* gene expression and phenotypic changes. A single clone was chosen for additional functional characterization. Compared with the parental A549 (control) cells, western blot analysis revealed a near total disappearance of SNX5 protein in the selected clone (Fig. 6a). Viable cell counting over 96 h showed the A549 control cells proliferation significant faster than the A549-SNX5-KO cells, with the final counts at 8.98 × 10^4^ ± 1.3 × 10^3^ viable cells in the control cell line and a decrease to 4.80 × 10^4^ ± 1.4 × 10^3^ viable cells in SNX5-KO cell line (Fig. 6b). Although the knockout of *SNX5* gene in tumor cells shows a reduction in cell proliferation, the cell migration and invasion abilities were largely maintained (Fig. 6e). Compared with the A549 control cells, A549-SNX5-KO cells exhibited significantly lower iATP levels (Fig. 6c), drastically reduced macropinocytosis (Fig. 6d), and reduced invasion (Fig. 6e). These assay results indicated, for the first time, that macropinocytosis was deeply involved in and significantly contributes to eATP-induced EMT, strongly suggesting an intracellular component in the mechanism of eATP-induced EMT.

### *SNX5* gene KO led to slower tumor formation and tumor growth in vivo

To evaluate *SNX5* gene KO on cell growth in vivo, parental A549 (control) cells and A549snx5ko cells were used to generate tumors in nude mice. Tumor volume was measured during the study and tumor weights were measured after mice were euthanized and tumors were removed. Compared to A549 tumors, A549snx5ko tumors were about one third of the average weight of the wild type tumors at the end of the study (Fig. 6f, g) and they also grew significantly slower during the study (Fig. 6h). Comparison of two tumor phenotypes shows that SNX5 KO tumors are not only smaller in size, but also lighter in color, and more regular in tumor shape (Fig. 6f) but no major morphological difference at cell level (Fig. 6i). These in vivo results directly and indirectly support our hypothesis that macropinocytosis plays very important roles in extracellular nutrients (including eATP) uptake and tumor growth and metastasis, as we previously showed A549 tumors internalized ATP by macropinocytosis [21].

**Fig. 7:**
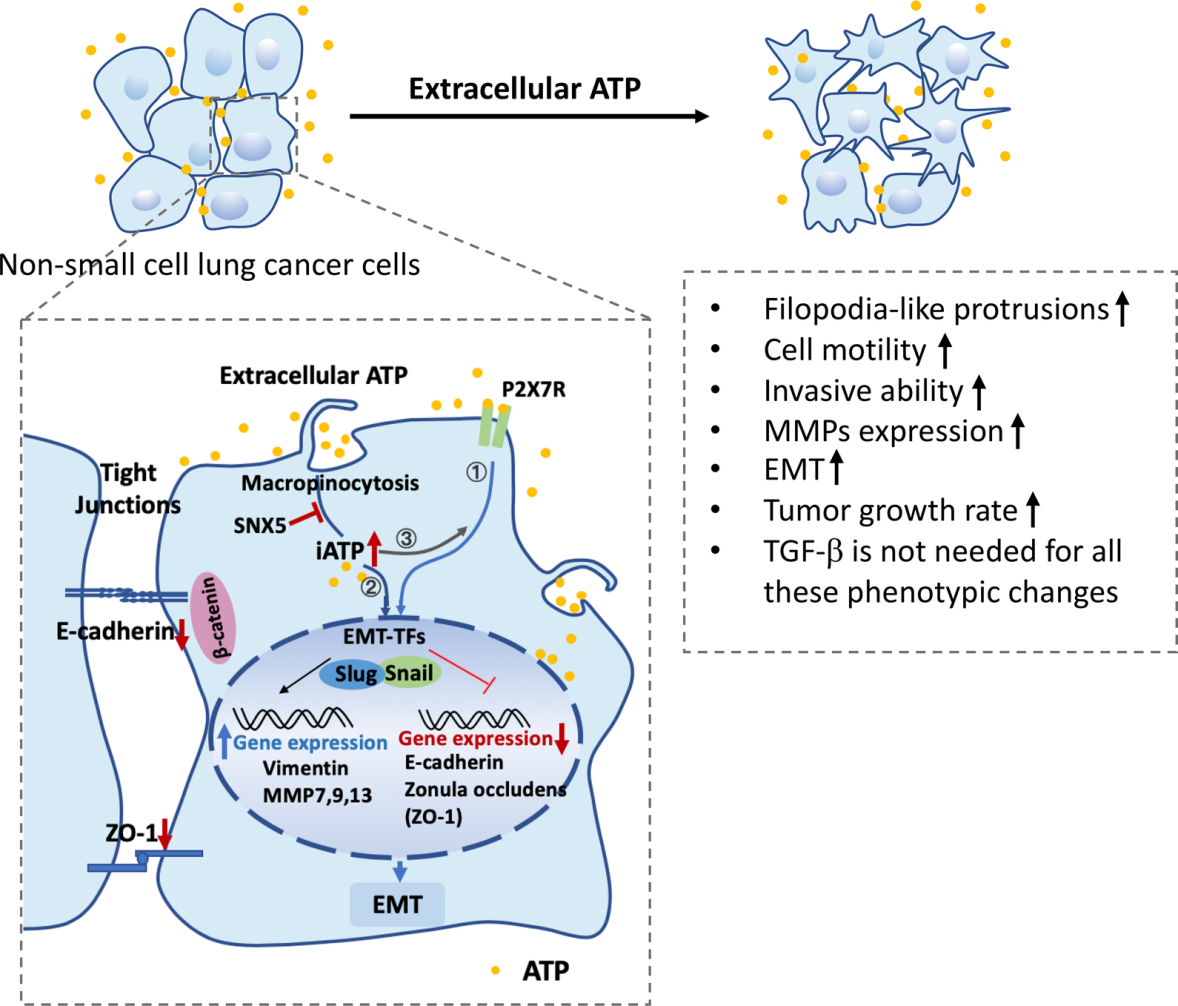
A hypothetical model for extracellular ATP-induced EMT, cell migration, and invasion. Extracellular ATP, as a messenger, binds and activates purinergic signaling via P2X7. P2X7 mediated signaling leads to EMT induction through upregulation of mesenchymal markers and downregulation of epithelial markers via enhanced transcription factors Snail and Slug and possibly others. eATP is also internalized primarily by macropinocytosis to significantly elevate intracellular ATP levels and further induce EMT. eATP, as a messenger or an energy molecule, also directly and indirectly accelerates cell detachment, migration, and invasion

Taking all data into consideration, we have generated a hypothetical model for eATP’s functions in EMT induction and in cell migration/invasion (Fig. 7).

## Discussion

ATP is one of the most conserved molecules in the bio-world that still surprises us with unexpected new functions in the 21st century [21, 22, 26]. In addition to being a “universal energy currency” and a phosphate donor, ATP is also an extracellular messenger [20, 27]. ATP is an omnipotent and omnipresent molecule inside and outside of cells, particularly in tumors.

Dysregulated energetics has been named as a new hallmark of cancer [28]. Opportunistic uptake of extracellular nutrients via macropinocytosis and other processes has been more recently named as the primary hallmark of cancer metabolism [29]. These new characterizations indicate the newly recognized importance of energy metabolism and macropinocytosis as a way of acquiring free energy and nutrients from outside of cancer cells. ATP, as one of the most important and abundant extracellular nutrients present in the tumor microenvironment (TME) at levels of 10^3^ to 10^4^ times higher than those in normal tissues [16–19], plays a central role in cancer energy metabolism and the well-known Warburg effect [30–32]. However, the sources, destinations, and functions of the eATP in the TME are far from fully known.

In this study, ATP was shown to induce relatively rapid detachment of cancer cells from the surface of cell culture dishes. The detached cells became floating and then moved to other parts of the dishes to form new colonies. All of these new findings are consistent with a proposed dynamic model for tumor growth [33], in which some cancer cells in the center region of the tumor are predicted to leave their original locations and move outward to form new clones on the outer surface of the tumor, generating a faster growing, multi-clonal, and multi-nodule tumor. However, this model does not specify factors responsible for the outward movement of the cancer cells. Our results provide a simplified 2-D model for how intratumoral eATP initiates and facilitates cell movement in a real 3-D tumor environment.

We also showed that eATP alone is sufficient to induce EMT, migration, and even invasion in cultured cancer cells. This further demonstrates ATP’s multi-functional features: as an extracellular energy source to drive cell detachment, a messenger to extracellularly activate purinergic receptor (P2X7)-mediated EMT induction, a phosphate donor to upregulate the EMT signaling from inside of the cancer cells (as we found in our previous studies) after macropinocytosis-mediated internalization, and an intracellular energy source to facilitate cell morphology change (formation of filopodia-like protrusions) and movement.

We further demonstrated that eATP not only induces EMT-related activities in NSCLC A549 cells, but also in NSCLC H1299 and Hop-92 cells (Figs. 1a, b; 2c, d; and Additional file 1: Figure S1c, d), indicating that eATP-induced EMT is a common phenomenon among NSCLC cell lines. This phenomenon may be prevalent among all cancer types that have an active P2X7 signaling pathway [7, 8, 10–12] and macropinocytosis that efficiently internalizes eATP and elevates iATP levels [20, 21]. Moreover, macropinocytosis is upregulated in KRas mutated cancer cells such as A549 cells [34–37], and therefore cancer cells with KRas mutations should be more sensitive to eATP’s induced EMT and cell movement.

Our previous studies showed that eATP is internalized primarily by macropinocytosis to elevate iATP levels, promote cell growth and survival [20, 21], and augment resistance to anticancer drugs [22]. Studies by others have shown that TGF-β induces EMT via exocytosis of ATP-filled vesicles and subsequent activation of purinergic receptor P2X7 by the released ATP (which is equivalent to eATP) [12, 13]. In this study, we have shown that eATP induces EMT both extracellularly and intracellularly. One of the eATP’s extracellular activities is activating P2X7 signaling. eATP’s intracellular activity is via macropinocytosis-mediated eATP internalization and the resulting increase in iATP concentration (Figs. 5 and 6). Furthermore, as an energy molecule and a phosphate donor that provides readily available energy, the elevated iATP is likely to accelerate biochemical reactions and upregulates signal transduction, including those involved in EMT, migration and invasion.

Purinergic receptor (PR) signaling, particularly P2X7 signaling, has been shown to play an essential role in TGF-β-mediated EMT induction [12–15, 38]. Our study has confirmed this result. Moreover, our study has further shown that eATP, at the concentration range of the reported intratumoral eATP [16–19], induced much more migration and invasion than TGF-β (Fig. 3g, h). It is conceivable that in real tumors, EMT and other metastatic steps can be induced by either intratumoral eATP or TGF-β, whichever is available in the TME at concentration ranges that are EMT- and metastasis-inducible, providing induction flexibility to cancer cells. The detailed relationship between TGF-β and eATP on metastasis remains to be delineated.

Macropinocytosis plays an increasingly recognized role in nutrient uptake in cancer [20–22, 29, 39–43]. SNX5 knockdown and knockout stopped most of the macropinocytosis in A549 cells, showed significant reduction in cancer cell proliferation, cell migration/invasion, and tumor growth (Fig. 6). Compared to the wild type tumors, SNX5 KO tumors are much smaller in size, lighter in color (indicative of less angiogenesis), and more regular in tumor shape. These morphological changes are consistent with a proposed tumor growth model [33], which predicts that cancer cells in a tumor constantly detach from their original locations and move outward to establish new colonies. The knockout of *SNX5* gene reduced macropinocytosis and thereby reduced extracellular nutrient internalization and EMT, leading to decreased tumor cells’ movement and reduced tumor shape irregularity in the KO tumors. These studies have confirmed the important roles of SNX5 in macropinocytosis, and macropinocytosis in eATP-mediated tumorigenesis and metastasis, as we previously showed that ATP was abundantly internalized by A549 tumors by macropinocytosis in the same nude mouse model [21]. As intratumoral eATP levels of A549 SNX5 KO tumors are likely to be 10^3^ to 10^4^ times higher than in those found in normal lung tissues, the blocking of eATP internalization by the *SNX5* knockout is likely to contribute to the dramatic changes observed in these KO tumors. This is the first time the KO of SNX5 is found to drastically affect human tumor growth in a nude mouse model. However, this animal study only indirectly correlates eATP with EMT in vivo. The final proof of the mechanism has to wait for the availability of a bioluminescence traceable tumor model.

Based on previous and present experimental evidence, we propose a hypothetical model for eATP’s mechanisms of action in induction of EMT and early steps of metastasis following EMT (Fig. 7). In this model, eATP functions extracellularly and intracellularly. Extracellularly, eATP functions as a messenger to activate P2X7-mediated signaling for EMT induction, an MMP expression inducer and an energy source for cell detachment. Intracellularly, internalized eATP via macropinocytosis elevates iATP levels, altering levels of EMT related proteins and inducing cell morphology changes and movement, as well as increasing rates of enzymatic reactions involved in these processes. The extracellular P2X7 activation by ATP was previously known [12, 13]. However, all macropinocytosis-mediated intracellular mechanisms and some of the cell detachment phenomena described here have never been reported before. Importantly and different from the conventional belief, all these changes are achieved by ATP alone and without involvement of TGF-β. These indicate eATP is involved in EMT and many other early steps of metastasis by performing a very wide variety of different functions. It is indeed a master regulator of EMT and metastasis.

In a recent review, six unresolved fundamental issues associated with the EMT program in cancer were proposed [4]. Extracellular ATP is likely to be an answer, at least partially, to some of these issues such as (a) the nature of the heterotypic signals that converge on cancer cells and collaborate to activate previously silent EMT programs in these cells and (b) the roles of intracellular and extracellular signaling pathways in sustaining the expression of already-activated EMT programs [4].

Finally, cancer cells do not seem to have a shortage of ATP in vivo [31, 44, 45] even though their mitochondrial oxidative phosphorylation (ATP synthesis) is limited by hypoxia. Our new findings provide a possible explanation to the key remaining questions related to the Warburg effect, such as how cancer cells grow faster than normal cells without the need for synthesizing more ATP. The presence of high levels of eATP in the TME and the prevalence of macropinocytosis and therefore eATP internalization among cancer cells appears to, at least in part, account for this puzzling observation. These newly identified mechanisms and contributing factors enable us to reconsider the omnipotent and omnipresent roles of ATP in cancer from cell growth, to drug resistance, and now various early steps of metastasis.

## Conclusion

We have found that eATP, alone, is sufficient to induce cell detachment, EMT, migration and invasion in several human lung cancer cell lines independent of TGF-β, which was traditionally considered to be important and necessary for EMT induction. Mechanisms used by eATP in EMT/metastasis induction, identified in this study, have a known extracellular component but also a previously unknown intracellular component, which involves internalization of eATP by macropinocytosis. *SNX5*, a macropinocytosis related gene, has been knocked out for the first time in human lung cancer cells and shown to be very important for tumor growth rate and tumor morphology in vivo, for the first time linking extracellular ATP with macropinocytosis in tumorgenesis and metastasis. These new findings call for reconsideration and rebalance of energy equations in cancer metabolism and the Warburg effect. eATP, in addition to its previously identified activities in cancer, appears to function as a master regulator of EMT and various steps of metastasis. Inhibiting eATP function and reducing eATP levels, therefore, are likely to significantly diminish EMT induction, slow down metastasis, and reduce cancer related death.

## Supplementary information

**Additional file 1: Figure S1.** Extracellular ATP induces motility and invasion of human NSCLC. **a-b** Effect of extracellular ATP on wound healing of A549 cells. Confluent NSCLC A549 cell monolayers growing in cell culture plates were wounded using a sterile pipette tip and then treated with 0 mM, 0.5 mM or 1.0 mM ATP. **a** Representative photographs were taken at 0 hr, 12 hrs and 24 hrs post-wound. **b** The wound closure was quantified at 0 hr, 12 hrs and 24 hrs post-wound by measuring the remaining unoccupied area. **c-d** Extracellular ATP induces motility and invasion in human NSCLC Hop-92 cells. Representative images and the quantitative analysis following **c** the migration and **d** invasion assays in Hop-92 cells.

## Data Availability

All data generated or analyzed during this study are included in this published article (and its additional information files).

## Abbreviations

eATP: extracellular ATP
ECM: extracellular matrix
EMT: epithelial to mesenchymal transition
NSCLC: non-small cell lung cancer
oATP: oxidized ATP
PR: purinergic receptor
SNX5: sorting nexin 5
TF: transcription factor
TME: tumor microenvironment

## References

[1] Mehlen P, Puisieux A (2016). Metastasis: a question of life or death. Nat Rev Cancer.

[2] Lambert AW, Pattabiraman DR, Weinberg RA (2017). Emerging biological principles of metastasis. Cell.

[3] Brabletz T, Kalluri R, Nieto MA, Weinberg RA (2018). EMT in cancer. Nat Rev Cancer.

[4] Lamouille S, Xu J, Derynck R (2014). Molecular mechanisms of epithelial–mesenchymal transition. Nat Rev Mol Cell Biol.

[5] Dongre A, Weinberg RA (2019). New insights into the mechanisms of epithelial-mesenchymal transition and implications for cancer. Nat Rev Mol Cell Biol.

[6] Jolly MK, Ware KE, Gilja S, Somarelli JA, Levine H (2017). EMT and MET: necessary or permissive for metastasis?. Mol Oncol..

[7] Heerboth S, Housman G, Leary M, Longacre M, Byler S, Lapinska K, Willbanks A, Sarkar S (2015). EMT and tumor metastasis. Clin Transl Med.

[8] Thiery JP (2002). Epithelial-mesenchymal transitions in tumour progression. Nat Rev Cancer.

[9] Gialeli C, Theocharis AD, Karamanos NK (2011). Roles of matrix metalloproteinases in cancer progression and their pharmacological targeting. FEBS J..

[10] De Craene B, Berx G (2013). Regulatory networks defining EMT during cancer initiation and progression. Nat Rev Cancer.

[11] Ye X, Weinberg R (2015). Epithelial-Mesenchymal Plasticity: a central regulator of cancer progression. Trend Cell Biol..

[12] Xu J, Lamouille S, Derynck R (2009). TGF-β-induced epithelial to mesenchymal transition. Cell Res.

[13] Katsuno Y, Lamouille S, Derynck R (2013). TGF-β signaling and epithelial-mesenchymal transition in cancer progression. Curr Opin Oncol.

[14] Nieto MA, Huang RY-J, Jackson RA, Thiery JP (2016). EMT: 2016. Cell.

[15] Takai E, Tsukimoto M, Harada H, Sawada K, Moriyama Y, Kojima S (2012). Autocrine regulation of TGF-β1-induced cell migration by exocytosis of ATP and activation of P2 receptors in human lung cancer cells. J Cell Sci.

[16] Pellegatti P, Raffaghello L, Bianchi G, Piccardi F, Pistoia V, Virgilio FD (2008). Increased level of extracellular ATP at tumor sites: in vivo imaging with plasma membrane luciferase. PLoS ONE.

[17] Wilhelm K, Ganesan J, Müller T, Dürr C, Grimm M, Beilhack A, Krempl CD, Sorichter S, Gerlach UV, Jüttner E, Zerweck A, Gärtner F, Pellegatti P, Di Virgilio F, Ferrari D, Kambham N, Fisch P, Finke J, Idzko M, Zeiser R (2010). Graft-versus-host disease is enhanced by extracellular ATP activating P2X7R. Nat Med.

[18] Michaud M, Martins I, Sukkurwala AQ, Adjemian S, Ma Y, Pellegatti P, Shen S, Kepp O, Scoazec M, Mignot G, Rello-Varona S, Tailler M, Menger L, Vacchelli E, Galluzzi L, Ghiringhelli F, di Virgilio F, Zitvogel L, Kroemer G (2011). Autophagy-dependent anticancer immune responses induced by chemotherapeutic agents in mice. Science.

[19] Falzoni S, Donvito G, Virgilio FD (2013). Detecting adenosine triphosphate in the pericellular space. Interface Focus.

[20] Qian Y, Wang X, Liu Y, Li Y, Colvin RA, Tong L, Wu S, Chen X (2014). Extracellular ATP is internalized by macropinocytosis and induces intracellular ATP increase and drug resistance in cancer cells. Cancer Lett.

[21] Qian Y, Wang X, Li Y, Cao Y, Chen X (2016). Extracellular ATP a new player in cancer metabolism: NSCLC cells internalize ATP in vitro and in vivo using multiple endocytic mechanisms. Mol Cancer Res MCR.

[22] Wang X, Li Y, Qian Y, Cao Y, Shriwas P, Zhang H, Chen X, Wang X, Li Y, Qian Y (2017). Extracellular ATP, as an energy and phosphorylating molecule, induces different types of drug resistances in cancer cells through ATP internalization and intracellular ATP level increase. Oncotarget.

[23] Di Virgilio F, Sarti AC, Falzoni S, De Marchi E, Adinolfi E (2018). Extracellular ATP and P2 purinergic signalling in the tumour microenvironment. Nat Rev Cancer.

[24] Lim JP, Gosavi P, Mintern JD, Ross EM, Gleeson PA (2015). Sorting nexin 5 selectively regulates dorsal-ruffle-mediated macropinocytosis in primary macrophages. J Cell Sci.

[25] Itai N, Shimazu T, Kimura T, Ibe I, Yamashita R, Kaburagi Y, Dohi T, Tonozuka T, Takao T, Nishikawa A (2018). The phosphorylation of sorting nexin 5 at serine 226 regulates retrograde transport and macropinocytosis. PLoS ONE.

[26] Patel A, Malinovska L, Saha S, Wang J, Alberti S, Krishnan Y, Hyman AA (2017). ATP as a biological hydrotrope. Science.

[27] Song S, Jacobson KN, McDermott KM, Reddy SP, Cress AE, Tang H, Dudek SM, Black SM, Garcia JGN, Makino A, Yuan JX-J (2016). ATP promotes cell survival via regulation of cytosolic [Ca2+] and Bcl-2/Bax ratio in lung cancer cells. Am J Physiol Cell Physiol.

[28] Hanahan D, Weinberg RA (2011). Hallmarks of cancer: the next generation. Cell.

[29] Pavlova NN, Thompson CB (2016). The emerging hallmarks of cancer metabolism. Cell Metab.

[30] Vander Heiden MG, Cantley LC, Thompson CB (2009). Understanding the warburg effect: the metabolic requirements of cell proliferation. Science.

[31] Chen X, Qian Y, Wu S (2015). The Warburg effect: evolving interpretations of an established concept. Free Radic Biol Med.

[32] Cairns RA, Harris IS, Mak TW (2011). Regulation of cancer cell metabolism. Nat Rev Cancer.

[33] Waclaw B, Bozic I, Pittman ME, Hruban RH, Vogelstein B, Nowak MA (2015). A spatial model predicts that dispersal and cell turnover limit intratumour heterogeneity. Nature.

[34] Karachaliou N, Mayo C, Costa C, Magrí I, Gimenez-Capitan A, Molina-Vila MA, Rosell R (2013). KRAS mutations in lung cancer. Clin Lung Cancer.

[35] Prior IA, Lewis PD, Mattos C (2012). A comprehensive survey of Ras mutations in cancer. Cancer Res.

[36] Lubeseder-Martellato C, Alexandrow K, Hidalgo-Sastre A, Heid I, Boos SL, Briel T, Schmid RM, Siveke JT (2016). Oncogenic KRas-induced increase in fluid-phase endocytosis is dependent on N-WASP and is required for the formation of pancreatic preneoplastic lesions. EBioMedicine.

[37] Yoon Y-K, Kim H-P, Han S-W, Oh DY, Im S-A, Bang Y-J, Kim T-Y (2010). KRAS mutant lung cancer cells are differentially responsive to MEK inhibitor due to AKT or STAT3 activation: implication for combinatorial approach. Mol Carcinog.

[38] Burnstock G, Di Virgilio F (2013). Purinergic signalling and cancer. Purinergic Signal..

[39] Commisso C, Davidson SM, Soydaner-Azeloglu RG, Parker SJ, Kamphorst JJ, Hackett S, Grabocka E, Nofal M, Drebin JA, Thompson CB, Rabinowitz JD, Metallo CM, Heiden MGV, Bar-Sagi D (2013). Macropinocytosis of protein is an amino acid supply route in Ras-transformed cells. Nature.

[40] Recouvreux MV, Commisso C (2017). Macropinocytosis: a metabolic ddaptation to nutrient stress in cancer. Front Endocrinol.

[41] Trajkovska M (2013). Macropinocytosis supports cancer cell proliferation. Nat Cell Biol.

[42] Davidson SM, Jonas O, Keibler MA, Hou HW, Luengo A, Mayers JR, Wyckoff J, Del Rosario AM, Whitman M, Chin CR, Condon KJ, Lammers A, Kellersberger KA, Stall BK, Stephanopoulos G, Bar-Sagi D, Han J, Rabinowitz JD, Cima MJ, Langer R, Vander Heiden MG (2017). Direct evidence for cancer-cell-autonomous extracellular protein catabolism in pancreatic tumors. Nat Med.

[43] Commisso C (2018). The pervasiveness of macropinocytosis in oncological malignancies. Philos Trans R Soc B.

[44] Koppenol WH, Bounds PL, Dang CV (2011). Otto Warburg’s contributions to current concepts of cancer metabolism. Nat Rev Cancer.

[45] Lunt SY, Vander Heiden MG (2011). Aerobic glycolysis: meeting the metabolic requirements of cell proliferation. Annu Rev Cell Dev Biol.

