# Supplementary figures and images for: Extracellular and macropinocytosis internalized ATP work together to induce epithelial–mesenchymal transition and other early metastatic activities in lung cancer
Source: Cancer Cell Int. 2019 Oct 1;19:254. doi: 10.1186/s12935-019-0973-0 (PMC6771108; doi:10.1186/s12935-019-0973-0)

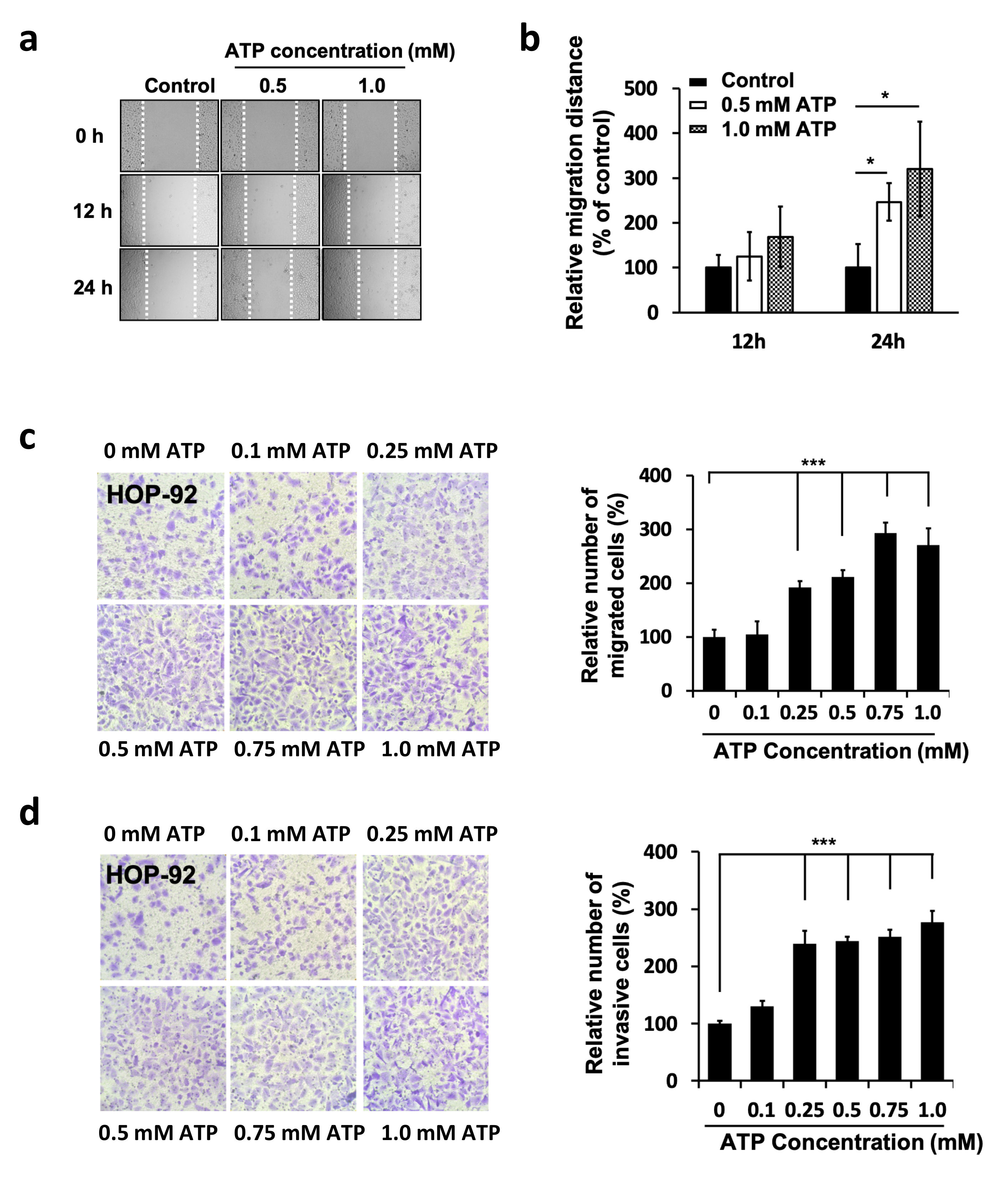

Supplement: Supplementary file 1 — Additional file 1: Figure S1. Extracellular ATP induces motility and invasion of human NSCLC. a-b Effect of extracellular ATP on wound healing of A549 cells. Confluent NSCLC A549 cell monolayers growing in cell culture plates were wounded using a sterile pipette tip and then treated with 0 mM, 0.5 mM or 1.0 mM ATP. a Representative photographs were taken at 0 hr, 12 hrs and 24 hrs post-wound. b The wound closure was quantified at 0 hr, 12 hrs and 24 hrs post-wound by measuring the remaining unoccupied area. c-d Extracellular ATP induces motility and invasion in human NSCLC Hop-92 cells. Representative images and the quantitative analysis following c the migration and d invasion assays in Hop-92 cells. [file 12935_2019_973_MOESM1_ESM.tif]
